# Neutrophil reprogramming underlie vasculopathy and lung disease in systemic sclerosis

**DOI:** 10.1038/s41419-026-08690-5

**Published:** 2026-04-04

**Authors:** Norma Maugeri, Giuseppe A. Ramirez, Annalisa Capobianco, Antonella Monno, Erika Arosio, Marco D’Ercole, Marco E. Bianchi, Patrizia Rovere-Querini, Florenzo Iannone, Marco Matucci-Cerinic, Angelo A. Manfredi

**Affiliations:** 1 Università Vita-Salute San Raffaele, Milano, Italy; 2 IRCCS San Raffaele Scientific Institute, Milano, Italy; 3 Charles River Italy RMS, Milano, Italy; 4 Department of Precision and Regenerative Medicine and Ionian Area, Rheumatology Unit University of Bari, Bari, Italy

**Keywords:** Translational research, Neutrophils

## Abstract

The role of neutrophils in systemic sclerosis (SSc) remains incompletely understood. To address this, blood samples from 39 SSc patients, 39 healthy controls, and 22 systemic lupus erythematosus (SLE) patients were analyzed. Our results indicate that in SSc, neutrophils exhibited substantial activation, evidenced by granule mobilization, elevated plasma levels of Neutrophil Extracellular Trap (NET) byproducts, and upregulated TIE2 expression. In parallel, they underwent metabolic reprogramming, characterized by increased autophagy, likely to support the heightened energy demands of activation. By contrast, neutrophils from SLE patients displayed minimal autophagy, lacked TIE2 expression, and shifted toward low-density granulocytes. Neutrophil reprogramming in SSc correlated with plasma levels of HMGB1^+^ EVs. Mechanistically, EVs purified from the plasma of patients with SSc adhered to neutrophils when injected in immunodeficient NSG mice, inducing autophagy, TIE2 expression, and promoting lung inflammation and fibrosis. These effects were abrogated by HMGB1 inhibitors and required the HMGB1 receptor, RAGE. Recombinant HMGB1 recapitulated EV-induced effects, while neutrophil targeting by liposome-encapsulated clodronate prevented them. In summary, neutrophils in SSc exhibit a dual phenotype of autophagy and activation driven by HMGB1^+^ EVs, representing a pathogenic mechanism with therapeutic potential in SSc. This mechanism operates similarly in male and female mice and is sufficient to induce neutrophil-driven lung injury in vivo.

## Introduction

Systemic sclerosis (SSc) is characterized by widespread vascular abnormalities and progressive fibrosis of the skin and internal organs. Therapeutic options remain limited, underscoring the need for translational research to bridge the gap between basic science discoveries and clinical applications [1, 2]. Early events in SSc natural history include endothelial cell activation and death, capillary loss, and exposure of sub-endothelial tissues. This results in progressive thickening of the media and hyperplasia of the intima, eventually leading to lumen obliteration [2].

Platelets activation and degranulation has been described across patient cohorts [3]. It contributes to microvascular damage and vessel wall remodeling [4–7] and is closely associated with neutrophil activation, which significantly influences gene expression in patients’ blood samples [3, 8, 9]. Normalization of neutrophils gene expression correlates with the improvement in lung function observed following hematopoietic stem cell transplantation [10]. Functional studies have also directly implicated neutrophils activation and functional exhaustion in the natural history of SSc [11, 12]. In two independent prospective SSc cohorts, blood neutrophil count and the neutrophil-to-lymphocyte ratio, which do not account for the heterogeneity of neutrophil composition, predicted an increased mortality and more severe disease [10]. Byproducts of NETs, that reflect neutrophil activation, accumulate in SSc plasma [7, 13–15] and are correlated with biomarkers of vascular injury, including soluble E-selectin and VCAM-1 [16], and with endogenous neutrophil agonists, such as mitochondrial-derived N-formyl methionine [17].

The mechanisms underlying neutrophil involvement and its association with vasculopathy remain elusive in SSc. In contrast, significant advancements over the past years have elucidated the multifaceted roles of neutrophils in other systemic autoimmune diseases. Notably, in systemic lupus erythematosus (SLE), neutrophils are well known to contribute to vascular dysfunction and homeostasis disruption, while fostering the autoimmune response by releasing oxidized nucleic acids and post-translationally modified histones into the extracellular space [18]. Furthermore, extracellular nucleic acids in SLE amplify type I interferon production by plasmacytoid dendritic cells, perpetuating the inflammatory cycle [18].

Extracellular vesicles (EVs) are membrane-bound structures released during cell activation or stress [7, 14, 19]. EVs accumulate in the blood of patients with SSc and are biologically active, contributing to key features of the disease such as lung fibrosis [15]. At least in part, their bioactivity depends on the ability to present HMGB1, a potent agonist, to neutrophils and vascular cells [7, 14, 20].

In this study, we show that EVs accumulating in the blood of SSc patients trigger neutrophil reprogramming, inducing autophagy, upregulation of the angiopoietin receptor TIE2, and the formation of NETs in a redox state-dependent manner. Furthermore, SSc EVs expressing HMGB1 induce in vivo lung fibrosis which can be prevented by depleting TIE2-expressing neutrophils. This may therefore emerge as a new potential treat to target pathway in SSc.

## Patients and methods

### Patients

The study group consisted of thirty-nine patients classified according to the 2013 ACR/EULAR criteria for SSc (29 females) [21] (see Table 1 for demographic and clinical characteristics). Thirty-nine age-matched healthy donors (median 51 years old, range 29–81; 21 females) served as controls. Twenty-two patients with SLE (17 females) classified according to the EULAR 2019 criteria [22] were studied in parallel. Patients with other systemic autoimmune disorders or overlap syndromes were excluded. All subjects gave their written informed consent to participate to the study. The study protocol was approved by the Ethics Committee of IRCCS San Raffaele Hospital (2/2013/INT, 124/INT/2016 and PNRR-MR1-2022-12376638), and written informed consent was obtained from all participants in accordance with the Declaration of Helsinki.

**Table 1:** SSc patients’ characteristics.

*N*	39
Sex, female/male	29/10
Age, median (25–75 percentile) years	54 (46–64)
**Disease characteristics**	
Disease duration, median (25–75 percentile) years	4 (2–15.5)
History of cardiovascular disease (*n*)	5
History of neoplasy (*n*)	1
Diffuse cutaneous SSc (%)	29
Limited cutaneous SSc (%)	71
Sicca syndrome (*n*)	8
Modified Rodnan skin score, median (25–75 percentile)	5.5 (4–14.5)
NVC (scleroderma pattern/unspecific findings)	27
Fingertip ulcers (*n*)	17
Pulmonary involvement:	
Pulmonary fibrosis (*n*)	8
Arterial pulmonary hypertension (*n*)	1
**Autoantibodies**	
Anti-nuclear antibodies (%)	89
Anti-topoisomerase I antibodies (%)	40
Anti-centromere antibodies (%)	46
Treatment	
Aspirin (*n*)	17
Tienopiridine (*n*)	4
Hydroxychloroquine (*n*)	4
Prednisone (*n*)	4
Dose median median (25–75 percentile)	5 (6.25–7.5)
Immune suppressive drugs (*n*)	5
Bosentan (*n*)	2

### Reagents

Monoclonal antibodies (mAbs) against CD45 (clone J33), CD61 (clone SZ21), CD66b (clone 80H3), CD62P (clone Thromb-6), PSGL-1 (clone PL-1), myeloperoxidase (MPO, clone CLB-MPO-1), relevant IgG isotype controls mAbs for flow cytometry, Thrombofix, Megamix, and Flow-Count™ Fluorospheres were obtained from Beckman Coulter (Italy). Monoclonal IgG1 isotype control (clone W3/25) was obtained from Acris Antibodies (Italy). mAb against HMGB1 (clone HAP-46.5), PGE1, Cell Death detection kit, glycated albumin, Thrombin Receptor Agonist Peptide-6 (TRAP-6), Sirius Red and Hoechst were obtained from Sigma (Italy). Anti-Collagen (Coll1A) from Santa Cruz (Italy). Recombinant human interleukin (IL)-8 was from R&D (Italy). Zenon IgG Labeling kits (488, 546 or 647) were obtained from Invitrogen. Rabbit polyclonal anti-histone H4 (citrulline R3¸ ab81797) was obtained from Abcam (Prodotti Gianni, Italy). mAb anti human MPO used for the determination of DNA-MPO complexes was from ABD Serotec. BoxA and recombinant HMGB1 were obtained from HMGBiotech (Italy). The CYTO-ID® Autophagy detection kit was obtained by Enzo Life Sciences (3 v Chimica, Milan, Italy). Monoclonal antibodies used to identify murine neutrophils (clone 7/4), and mAb anti RAGE (ab54741) antibodies were obtained from Abcam (Prodotti Gianni, Italy). Liposomes containing clodronate and liposomes containing PBS were obtained from ClodronateLiposomes.org (The Netherlands). mAb against TIE2 (clone 83715) for flow cytometry was from R&D (Italy) while the mAb against TIE2 (clone Ab33) for confocal and for electron microscopy was from Millipore (Italy). Low molecular weight Heparin (LMWH, parnaparin) was obtained from Alfasigma S.p.A. (Italy).

### Blood sampling

Venous blood was drawn through a 19-gauge butterfly needle. After having discarded the first 3–5 mL, blood was carefully collected in tubes containing either EDTA to purify platelets, neutrophils, PBMCs and EVs and to assess the concentration of NETs byproducts, or Na citrate and antiproteases for determination of cellular markers by flow cytometry [14, 23, 24].

### Human blood cell preparations

Platelets, neutrophils and platelet EVs suspensions were prepared as described [7, 14, 20, 24–27]. Briefly, blood samples were centrifuged at 150 × *g*, 10 min at 20 °C, to obtain platelet-rich plasma to be used to isolate platelets while the remaining blood was used to purified neutrophils (see below). Briefly, platelet rich plasma was recovered and PGE1 (2.5 µM) was added before centrifugation at 800 × *g* for 15 min at 18 °C (without break). Platelet pellets were washed twice with Hepes Tyrode buffer (129 mmol/L NaCl, 9.9 mmol/L NaHCO3, 2.8 mmol/L KCl, 0.8 mmol/L KH2PO4, 5.6 mmol/L dextrose, 10 mmol/L HEPES, MgCl2 1 mM, pH 7.4) containing NaEDTA (5 mM) and in the first wash PGE1 (2.5 µM) and resuspended with Hepes Tyrode buffer containing CaCl_2_ (1 mM).

#### Extracellular vesicles

EVs were isolated from EDTA anticoagulated plasma samples and characterized as previously described [7, 14]. Briefly, blood samples were centrifuged 15 min at 2000 × *g* at RT. Retrieved platelet-poor plasma was further centrifuged 15 min at 2000 × *g*, 4 °C to obtain platelet free plasma, then further centrifuged at 100,000 × *g* for 45 min at 4 °C. EVs were retrieved in the pellets and resuspended in Hepes Tyrode buffer containing CaCl_2_ and MgCl_2_ (both 1 mM final concentration). EV were also purified from the supernatant of purified platelets (5 × 10^5^/µL) stimulated with collagen (5 µg/mL) for 60 min at 37 °C in static conditions, then placed in chilled water bath for 2 min and centrifuged 15 min at 2000 × *g*, 4 °C. Supernatants were retrieved, centrifuged 45 min at 100,000 × *g*, 4 °C, pellets were resuspended in Hepes Tyrode buffer, and EVs expressing HMGB1 (HMGB1-EVs) were quantified by flow cytometry using flow count and diluted to a final concentration of 10^5^ HMGB1-EVs/µL.

EVs were characterized using a validated, multimodal pipeline developed and described in detail [28]. The EV isolation protocol was rigorously benchmarked using Western blotting for canonical EV markers, including CD9, CD63, CD81, and TSG101, and confirmed to be free of cellular contaminants. These results established the specificity and reproducibility of our protocol for isolating plasma-derived EVs. In the present study, we confirmed EV identity and integrity using transmission electron microscopy (TEM) for morphology (Supplementary Fig. 2), particle size analysis (Supplementary Fig. 3), and flow cytometry for phenotypic profiling. Fluorescent antibodies against CD61, HMGB1, P-selectin, and Tissue Factor were used to assess EV origin and surface marker expression, with a focus on platelet-derived vesicles, which are predominant in the context of SSc.

Although the full study cohort comprised 39 SSc patients and 39 healthy controls, some experimental procedures were carried out on slightly smaller subsets due to technical limitations, such as insufficient sample volume or suboptimal EV recovery. These cases were randomly distributed across groups and are explicitly detailed in the figure legends.

#### Neutrophils

Neutrophils were isolated from the remaining blood (after the first centrifugation to obtain PRP) as previously described [29] by Dextran sedimentation followed by Ficoll-Hypaque gradient and hypotonic lysis of erythrocytes, were washed and resuspended in HEPES-Tyrode buffer with 1 mM CaCl2.

#### Low density neutrophils

Frozen PBMCs suspensions were thawed, washed and fixed as previously described [30]. After 4 h at 4 °C, samples were labeled with anti CD45 and anti CD66b and analyzed by flow cytometry to identify neutrophils within mononuclear cells.

### Flow cytometry

Samples were analyzed on a diary aligned Navios flow cytometer (Beckman Coulter, Milan, Italy). Whole blood samples for the determination of cellular markers of activation were immediately fixed with equal volumes of Thrombofix, stored at 4 °C and analyzed within 6–24 h. Extent of platelet and leukocyte activation were assessed by multi parametric flow cytometry as previously described [20, 24, 27]. Quantification of platelet-derived EVs (plt-EVs) in platelet-free plasma or in the supernatant of purified stimulated platelets was performed as described [7, 14, 26].

### Neutrophil-EV interaction studies

Neutrophils freshly purified from the peripheral blood (5 × 10^3^/µL) were resuspended in Hepes Tyrode with 1 mM CaCl_2_. Neutrophils were challenged at 37 °C for 5 min with SSc- HMGB1^+^ plt-EVs, or with HMGB1^+^ plt-EVs, with recombinant HMGB1, with IL-8 or with glycated albumin. When indicated, plt-EVs were pretreated with blocking mAb against P-selectin, before the coincubation with neutrophils. Alternatively, neutrophils were pretreated with anti-PSGL-1 mAbs or with anti-RAGE before the incubation with platelets and plt-EVs. An irrelevant isotopic mAb was used as control. Reactions were stopped by addition of an equal volume of Thrombofix and samples stored at 4 °C until analysis.

### Mice

Ten- to twelve-week-old (24 female and 23 male) NOD.Cg-PrkdcscidIl2rgtm1Wjl/SzJ mice (NSG) and *Rage*^-/-^ mice were obtained from Charles River (Milano). Neutrophils were purified from the bone marrow by flushing femurs and tibiae with ice-cold buffer containing hirudin (15 μg/mL), EDTA (5 mM), and EGTA (5 mM). The suspension was filtered, and erythrocytes were eliminated by hypotonic shock. Neutrophils were isolated by histopaque gradient and resuspended in HEPES Tyrode’s containing CaCl2 (1 mM) and MgCl2 (1 mM). Purity of neutrophil preparations (consistently > 97%) was routinely verified with flow cytometry, and cell viability (>97%) was verified by trypan blue exclusion test. Nuclear morphology was verified after staining with Turk solution [7, 23]. Experiments conducted in both male and female mice did not reveal sex-related differences in neutrophil infiltration or lung fibrosis following EV injection. On this basis, and in line with ethical guidelines aimed at minimizing animal use and variability, subsequent experiments were balanced toward male mice.

Unless otherwise indicated, each experimental group included 4–6 mice. All animal procedures were approved by the Institutional Animal Care and Use Committee (IACUC approval number 1216 and 1371) of the same institution and conducted in compliance with EU Directive 2010/63 and relevant national regulations governing the use of laboratory animals.

### In vivo effects of EVs

EVs were injected in the tail vein of NSG mice (10^7^ EVs/mouse), as described [7, 14]. Sham-treated mice were injected with Hepes Tyrode CaCl2 1 mM. When indicated EVs were injected in the presence of HMGB1 inhibitors such as Box A (1 μg/mouse), LMWH or monoclonal anti-HMGB1 antibodies (clone HAP-46.5, 1 ug/mouse).

After 18 hs the blood was obtained from the orbital vein of the mice and the concentration of NETs byproducts (citrullinated Histone 4 and MPO-DNA complexes) and of plasma E-selectin assessed by ELISA [7, 14]. Neutrophil stunning was obtained by treatments with liposomes containing clodronate (ClodronateLiposomes.org, The Netherlands) before EVs injection. Mice injected with liposomes containing PBS served as controls [7].

### NETs quantification

MPO-DNA complexes as well as citrullinated Histone 4 were identified using capture ELISA as previously described [7, 14, 20, 23].

### Histochemistry

Lungs were isolated and immediately fixed in 4% paraformaldehyde at 4 °C for 6 h. Then placed in 10%, 20% and 30% sucrose, tissue sinks embedded in OCT and stored at −80 °C. 5 µm thick lung slices analyzed as previously described [7, 14, 23]. For fibrosis, collagen deposition was quantified by thresholding Congo red–positive areas and calculating the percentage of collagen-positive area relative to total tissue area in each section. Histological images Sirius red–stained lung sections were analyzed using ImageJ (NIH).

### Statistics

Results were reported as mean ± standard error of the mean (SEM), unless otherwise indicated. Kruskal-Wallis test followed by Dunn’s multiple comparison test was used to compare continuous variables between groups. All tests were two-sided and *p* values lower than 0.05 were considered statistically significant. In all analyses GraphPad Prism 10.3.1 was used.

## Results

### Autophagy and activation of neutrophils of patients with SSc

We compared neutrophils in the blood of patients with SSc to those of healthy volunteers. As a reference group, we studied patients with SLE, a disease in which dysregulated neutrophil activation plays a role in vascular disease [31]. However, unlike SSc, fibrosis and obliterative vasculopathy are uncommon in SLE. Tables 1 and 2 report the main characteristics of the patients.

**Table 2:** SLE patients’ characteristics.

*N*	22
Sex, female/total	17/22
Age, median (25–75 percentile) years	42 (33–52.3)
**Disease characteristics**	
Disease duration, median (25–75 percentile) years	14 (6–22.5)
SLEDAI, median (25–75 percentile)	2 (2–6)
Renal involvement (*n*)	11
Hematological involvement (*n*)	12
Neurological involvement	3
Antiphospholipid syndrome (*n*)	1
**Autoantibodies**	
Anti-ds DNA	19
Anti-Sm	5
Anti-phospholipid	10
**Treatment**	
Aspirin (n)	5
Tienopiridine (n)	1
Warfarin (n)	2
Hydroxychloroquine (n)	11
Prednisone (n)	5
Dose: median (25–75 percentile)	5 (2.5–5)
Methotrexate (n)	3
Mycophenolate mofetil	4

**Fig. 1:**
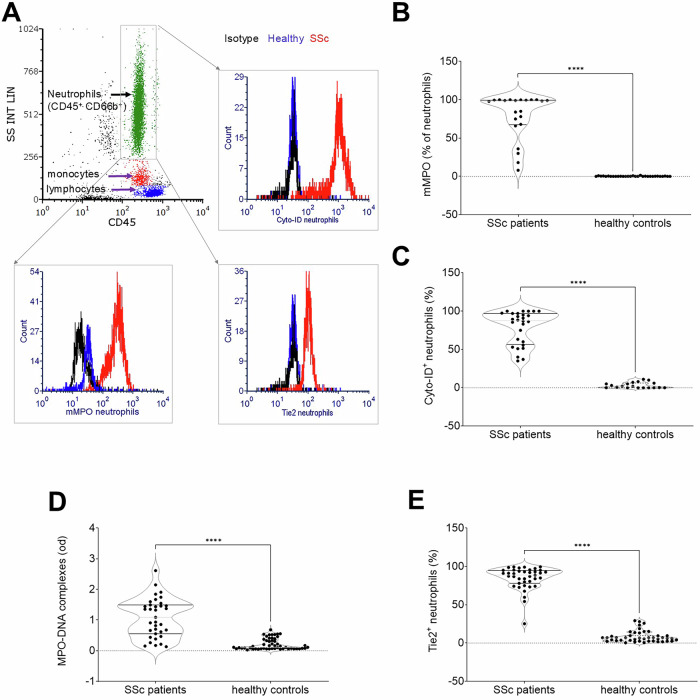
Neutrophil activation in systemic sclerosis. **A** Neutrophils were identified among blood leukocytes by flow cytometry, based on side scatter (SS), and the expression of the pan-leukocyte marker CD45 alongside the neutrophil-specific marker CD66b. Representative flow cytometry profiles shown in (**A**) illustrate that in SSc patients (red profiles), neutrophils exhibited significant autophagy, indicated by Cyto-ID dye accumulation in autophagosomes (SSc patients *n* = 29, healthy controls *n* = 20), displayed surface expression of MPO (SSc patients *n* = 22, healthy controls *n* = 31). Results for neutrophils from representative healthy controls (blue profiles) and with isotype-matched control antibodies (black profiles) are also shown. Comparison between SSc patients and healthy controls reveals significantly increased surface expression of MPO (**B**), elevated autophagy (assessed by Cyto-ID dye accumulation, **C**), enhanced NET generation (quantified by MPO-low molecular weight DNA complexes, **D**) (SSc patients *n* = 35; healthy controls *n* = 39), and heightened expression of the activation marker TIE2 in neutrophils from SSc patients (**E**) (SSc patients *n* = 39, healthy controls *n* = 39). Each point represents a single patient or healthy control. ****, significantly different from healthy controls, *p* < 0.001.

Most blood neutrophils in SSc patients, but not in healthy donors, were activated, as indicated by the localization of MPO to the plasma membrane, a bona fide evidence of azurophilic granules mobilization (Fig. 1A, B). Additionally, most neutrophils were autophagic, as assessed by the accumulation of the Cyto-ID tracer within autophagosomes (Fig. 1A, C), a feature linked to prolonged survival and extended biological action.

Autophagy also provides the necessary energy to produce NETs [32–34]. Indeed, complexes of low molecular weight DNA fragments with MPO, a marker for NET formation, were significantly more concentrated in the plasma of SSc patients (Fig. 1D). Among the markers indicating neutrophil activation, we assessed the expression of the angiopoietin receptor, TIE2, that has been involved in neutrophil activation and production of NETs [35, 36].

In SSc patients, a significant proportion of neutrophils exhibited elevated TIE2 expression on their plasma membranes (84.7 ± 2.4%) compared to healthy donors (8.3 ± 1.1%; *p* < 0.0001) (Fig. 1E). Immunogold electron microscopy further confirmed TIE2 localization within extra-granular cytoplasmic domains in SSc neutrophils, contrasting with its prevalent localization on the plasma membrane in neutrophils from healthy donors (Supplementary Fig. 1).

**Fig. 2:**
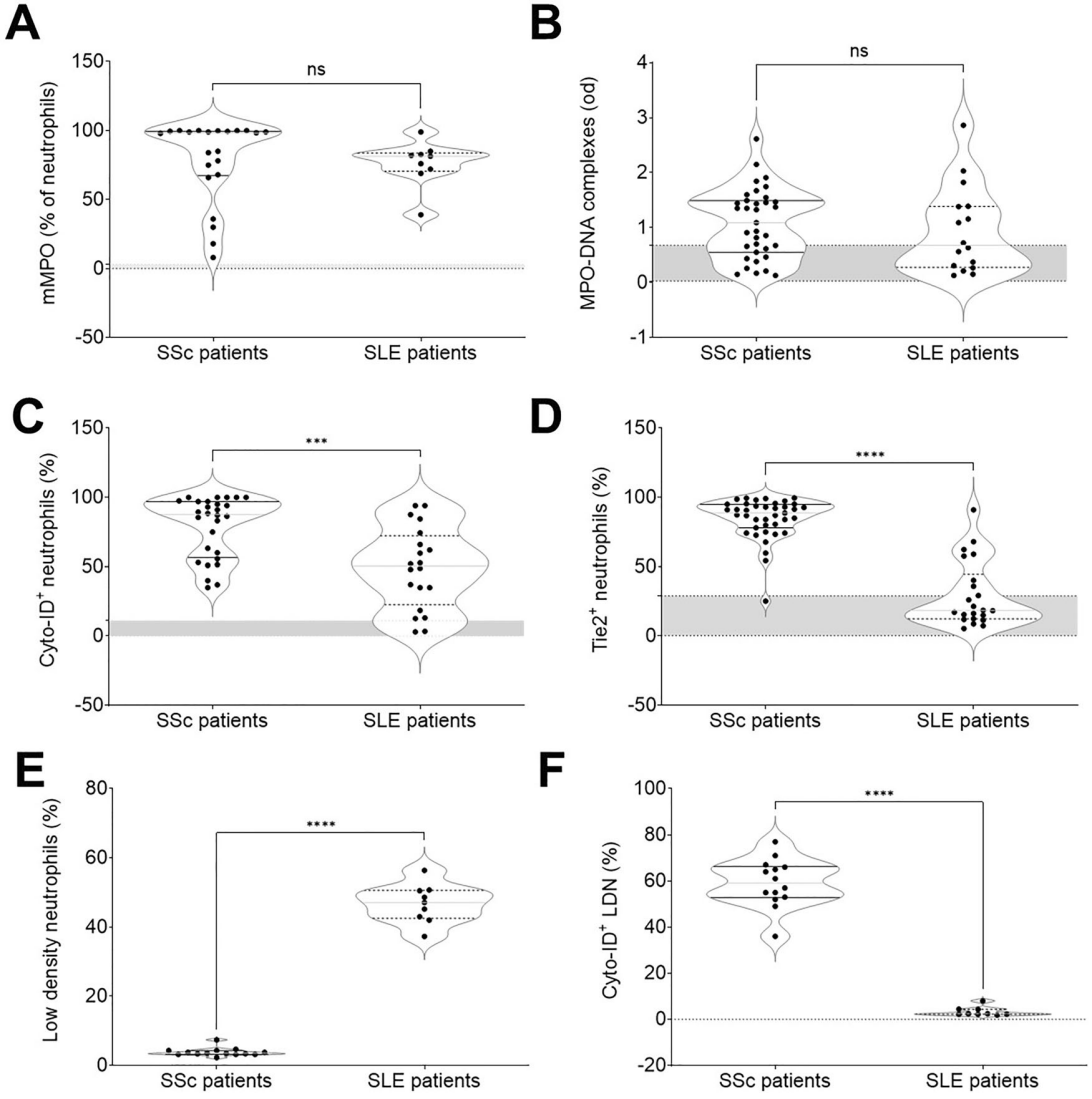
Different features of neutrophils activation in SLE and SSc. Comparison of neutrophil activation Comparison of neutrophil activation between patients with systemic sclerosis (SSc) and systemic lupus erythematosus (SLE) reveals similar levels of primary granule content mobilization (SSc *n* = 22, SLE *n* = 9), as indicated by membrane expression of myeloperoxidase (mMPO, **A**), and comparable neutrophil extracellular trap (NET) generation, quantified by MPO–low molecular weight DNA complexes (**B**). However, SSc patients exhibited significantly higher autophagy (assessed by Cyto-ID dye accumulation, **C**; SSc *n* = 28, SLE *n* = 19) and greater expression of the activation marker TIE2 (**D**; SSc *n* = 38, SLE *n* = 22). Additionally, low-density neutrophils (LDNs) were less abundant in SSc patients compared to SLE (**E**; SSc *n* = 13, SLE *n* = 8). Notably, in SSc, LDNs were autophagic, whereas in SLE these cells showed no signs of autophagy (**F**; SSc *n* = 13, SLE *n* = 8). Each point represents a single patient or healthy control. The gray area represents the minimum and maximum values observed in healthy controls. ** Significantly different from healthy controls, *p* < 0.001; *** significantly different from SLE patients, *p* < 0.005; ns non-significant.

Neutrophils from SLE patients also exhibited enhanced MPO membrane expression while NETs fragments accumulated in the patients’ plasma (Fig. 2A, B). Healthy controls were included as a reference group (shown in gray), confirming that both patient groups significantly differed from baseline values. However, neutrophils in SLE and SSc patients were markedly different: the extent of neutrophil autophagy and the proportion of TIE2-expressing neutrophils were significantly lower in SLE patients (Fig. 2C, D). As previously reported [37, 38], low-density neutrophils (LDNs) were a prominent subset within the peripheral blood mononuclear cell fraction isolated from SLE patients using density gradient centrifugation. In stark contrast, LDNs constituted only a minority of the mononuclear cells in SSc patients (Fig. 2E). Notably, the LDNs from SSc patients were autophagic, a feature absent in LDNs from SLE patients.

Given the neutrophil short half-life, it is likely that the stimulus driving their reprogramming persists in the SSc patient’s bloodstream. We have previously observed that HMGB1^+^ EVs trigger neutrophil activation, promote autophagy, and induce the production of NETs [7, 14, 23, 34].

**Fig. 3:**
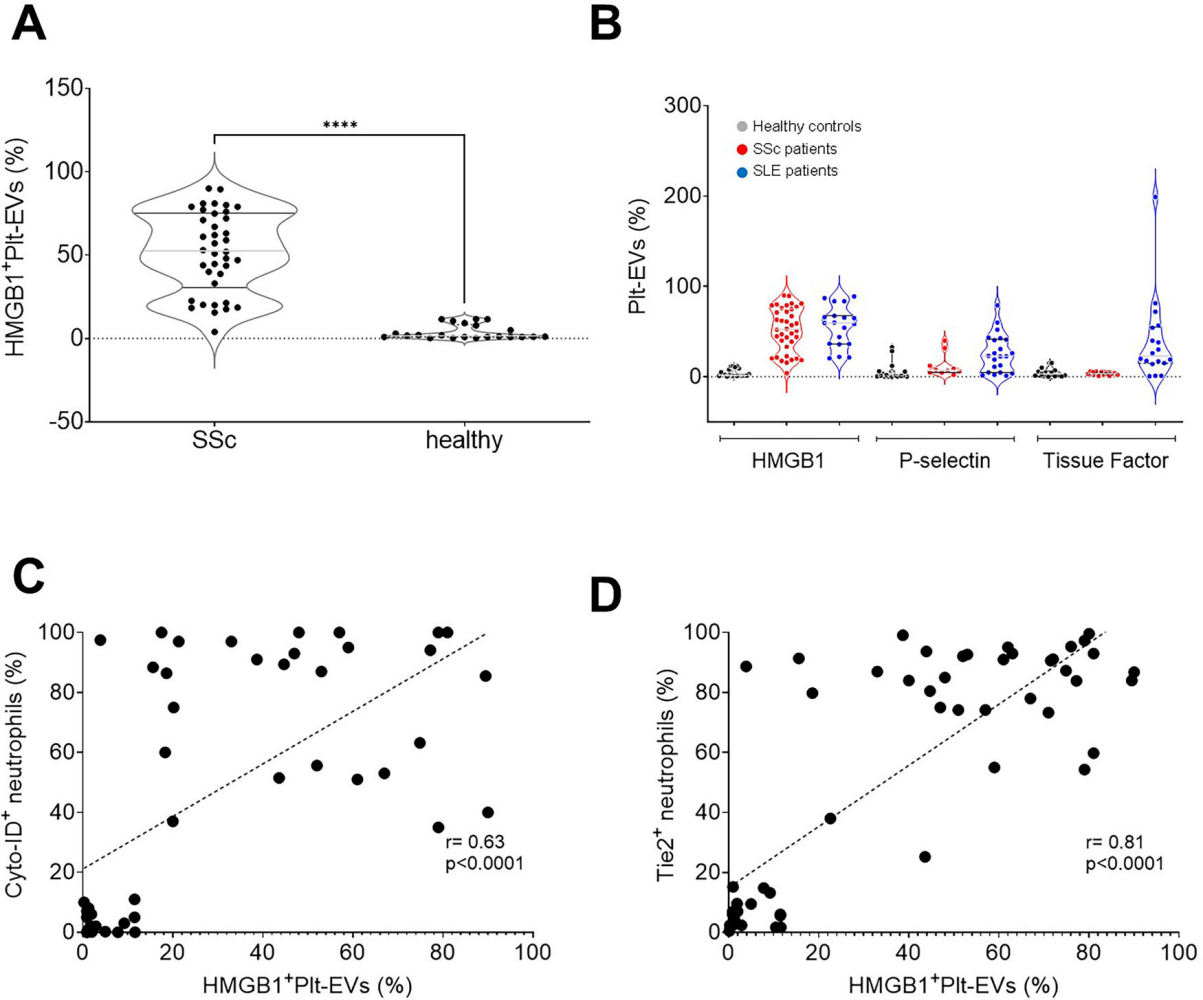
Accumulation of platelet HMGB1+ EVs correlates with neutrophil activation in SSc. Platelet-derived HMGB1⁺ extracellular vesicles (HMGB1⁺ plt EVs) were significantly more abundant in the blood of SSc patients (*n* = 38) compared to healthy controls (*n* = 21) (**A**). In contrast, there were no significant differences in platelet-derived EVs expressing P-selectin (SSc *n* = 10; SLE *n* = 12; healthy controls *n* = 20) or tissue factor (SSc *n* = 12; SLE *n* = 18; healthy controls *n* = 13) (**B**). HMGB1⁺ plt EVs (x-axis) showed a significant positive correlation with both neutrophil autophagy (**C** y-axis; *n* = 46) and TIE2 expression (**D** y-axis; *n* = 57). ****, *p* < 0.001.

HMGB1^+^ platelet-derived EVs (plt-EVs), identified based on the expression of the platelet lineage marker CD61 (GPIIbIIIa), were more concentrated in the blood of SSc patients compared to controls (*p* < 0.0001, Fig. 3A), and their levels significantly and strongly correlated with the expression of the TIE2 receptor on activated neutrophils and with the extent of autophagy (Fig. 3C, D), supporting the possibility of a causal link. In contrast, other signals potentially involved in EV interactions with the innate immune system, particularly with neutrophils—such as P-selectin—or in the activation of the coagulation system, like tissue factor were not significantly overexpressed on SSc EVs (Fig. 3B) and the concentration of P-selectin^+^ or TF^+^ EVs showed no correlation with the extent of neutrophil activation or autophagy.

Figure 3B enables a direct comparison of HMGB1, P-selectin, and Tissue Factor expression on CD61⁺ plt-EVs among patients with SSc, SLE, and healthy controls. Notably, SLE patients exhibited detectable levels of HMGB1 expression on plt-EVs. However, unlike in SSc, HMGB1 levels in SLE did not correlate with TIE2 expression or autophagy in neutrophils (*r* = 0.32, *p* = 0.18), suggesting a qualitative difference in HMGB1-mediated activation of neutrophils or the presence of disease-specific factors modulating its functional impact.

### Vesicular HMGB1 causes neutrophil TIE2 upregulation

**Fig. 4:**
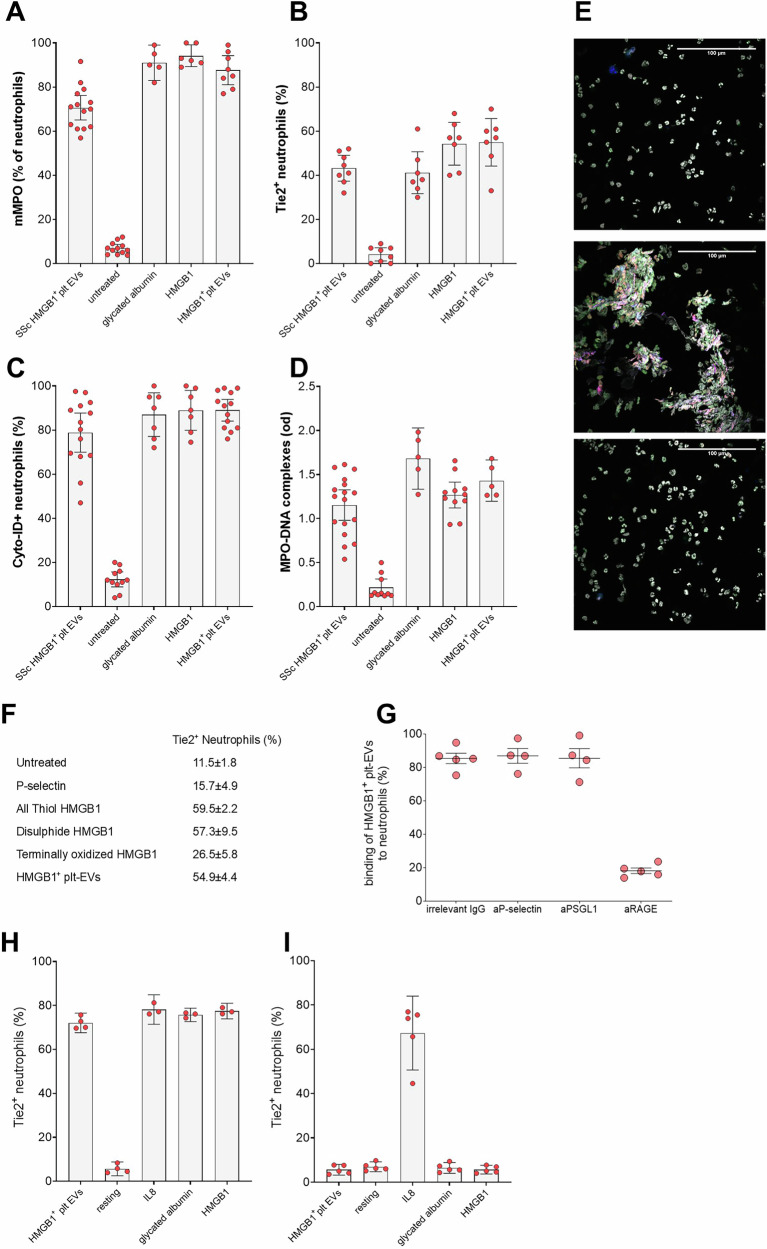
HMGB1+ EVs induce neutrophil activation through a RAGE-dependent pathway. Purified human neutrophils were either challenged with platelet-derived HMGB1⁺ EVs from the plasma of SSc patients (SSc HMGB1⁺ plt EVs), left untreated, or stimulated with the RAGE agonist glycated albumin, recombinant HMGB1, or HMGB1⁺ EVs from activated platelets (HMGB1⁺ plt EVs). Neutrophil activation was assessed by evaluating: membrane-bound MPO (mMPO, **A**) (SSc EVs *n* = 14; untreated *n* = 12; glycated albumin *n* = 5; HMGB1 *n* = 6; HMGB1⁺ EVs *n* = 8); expression of the activation marker TIE2 (**B**) (SSc EVs *n* = 8; untreated *n* = 8; glycated albumin *n* = 7; HMGB1 *n* = 7; HMGB1⁺ EVs *n* = 7); extent of autophagy measured by Cyto-ID dye accumulation (**C**) (SSc EVs n = 14; untreated *n* = 11; glycated albumin *n* = 7; HMGB1 *n* = 7; HMGB1⁺ EVs *n* = 14); and NET generation quantified as MPO–DNA complexes in the supernatant (**D**) (SSc EVs *n* = 17; untreated *n* = 10; glycated albumin *n* = 5; HMGB1 *n* = 11; HMGB1⁺ EVs *n* = 5). **E** Neutrophils were either left untreated (top panel) or exposed to platelet-derived HMGB1⁺ EVs in the absence (middle panel) or presence (bottom panel) of a monoclonal antibody blocking RAGE. Cells were analyzed by confocal microscopy for autophagy (Cyto-ID dye, green), TIE2 (Alexa 647, blue), and citrullinated histone H4 (Alexa 546, red). DNA was counterstained with Hoechst (white). **F** Neutrophils were either left untreated or exposed to purified human P-selectin, redox isoforms of HMGB1 (all-thiol, disulfide, or terminally oxidized), or platelet-derived HMGB1⁺ EVs (*n* **=** 5). TIE2 expression was assessed by flow cytometry. **G** Neutrophils were treated with isotype-matched control antibodies (*n* **=** 5) or antibodies specific for P-selectin (aP-selectin, *n* **=** 4), its counter-receptor PSGL1 (aPSGL1, *n* **=** 4), or the HMGB1 receptor RAGE (aRAGE, *n* **=** 5). The interaction between platelet-derived HMGB1⁺ EVs and neutrophils (y-axis) was measured by flow cytometry. Neutrophils from wild-type (**H**) and RAGE-deficient (**I**) mice were exposed to platelet-derived HMGB1⁺ EVs (**H** *n* **=** 4; **I** *n* **=** 5), left untreated (resting; **H** *n* **=** 4; **I** *n* **=** 5), or challenged with IL-8 (**H** *n* **=** 3; **I** *n* **=** 5), glycated albumin (**H** *n* **=** 3; **I** *n* **=** 5), or recombinant HMGB1 (H *n* **=** 3; **I** *n* **=** 5). TIE2 receptor expression on neutrophils was assessed by flow cytometry. Each point in the graphs represents an individual patient, healthy donor, or mouse.

To elucidate the causal relationship between EVs and neutrophil activation in SSc, we exposed normal neutrophils to EVs isolated from the plasma of SSc patients. Given that platelets are a primary source of EVs in SSc patients [7, 14, 39], we also generated EVs in vitro by activating platelets. Healthy neutrophils challenged with SSc EVs reproduced most features observed in neutrophils of SSc patients, including MPO expression on the neutrophil plasma membrane, expression of the TIE2 receptor, and accumulation of Cyto-ID dye in autophagosomes (Fig. 4A–C). Activation of neutrophils could also be detected by assessing the generation of NETs by autophagic neutrophils, either by measuring the concentration of MPO-DNA complexes in the supernatant or by fluorescence microscopy (Fig. 4D, E).

As a comparator, we also exposed neutrophils to EVs isolated from the plasma of SLE patients. However, these EVs failed to induce similar responses. Specifically, as shown in Table 3, SLE-derived EVs did not significantly promote autophagy, granule mobilization, NET release, or endothelial activation in vivo. This functional disparity supports the notion that HMGB1⁺ EVs in SSc possess disease-specific bioactivity, capable of reprogramming neutrophils in a way not observed with EVs from other autoimmune conditions.

**Table 3:** HMGB1-Positive Extracellular Vesicles from SSc Patients cause the In Vivo Activation of Neutrophils and Endothelial Cells.

	Autophagy (Cyto-ID + , % of neutrophils)	Granules mobilization (mMPO, % of neutrophils)	NETs (plasma citH4, OD)	Endothelial activation (plasma sE-selectin, ng/mL)
Sham-treated	16 ± 5	11 ± 1	0.07 ± 0.03	0.8 ± 0.06
SSc-EV	62 ± 4*	78 ± 2*	0.49 ± 0.05*	224 ± 43.5*
SSc-EV + HMGB1 blockade	11 ± 3	14 ± 2	0.07 ± 0.01	4.5 ± 1.2
SLE-EV	27 ± 6	18 ± 6	0.07 ± 0.02	2.0 ± 1.2
SLE-EV + HMGB1 blockade	38 ± 13	19 ± 3	0.09 ± 0.02	1.4 ± 0.6

^*^p < 0.001, significantly different from mice treated with saline (sham-treated, *n* = 3), from mice treated with SSc-EV (*n* = 3) in the presence of HMGB1 blockade with the competitive antagonist, BoxA, and from mice treated with SLE-EV (*n* = 3).

Purified recombinant human HMGB1 indeed reproduced the effects of the EVs purified from SSc patients, as did glycated albumin, a known agonist of the HMGB1 receptor RAGE (Fig. 4A), suggesting that the HMGB1-RAGE interaction may be involved. Indeed, interference with RAGE effectively abrogated the interaction of HMGB1^+^ plt-EVs with neutrophils, while blocking P-selectin or the PSGL1 counter-receptor on neutrophils did not affect plt-EV adhesion (Fig. 4F). The action of recombinant HMGB1 in triggering neutrophil TIE2 upregulation was dependent on the molecule’s redox state (Fig. 4G). Notably, purified P-selectin alone was ineffective at modulating TIE2 expression in neutrophils (Fig. 4G).

Additionally, HMGB1^+^ plt-EVs did not induce TIE2 expression in mouse neutrophils lacking the HMGB1 receptor, RAGE (Fig. 4H, I). Neutrophils from *Rage*⁻/⁻ mice upregulated TIE2 in response to the RAGE-independent agonist IL-8, demonstrating their ability to undergo activation. However, unlike wild-type neutrophils, *Rage*⁻/⁻ neutrophils failed to upregulate TIE2 when exposed to recombinant HMGB1 or the other RAGE agonist, glycated albumin (Fig. 4H, I). This indicates that RAGE is necessary for mediating plt-EVs HMGB1’s effects on neutrophils.

**Fig. 5:**
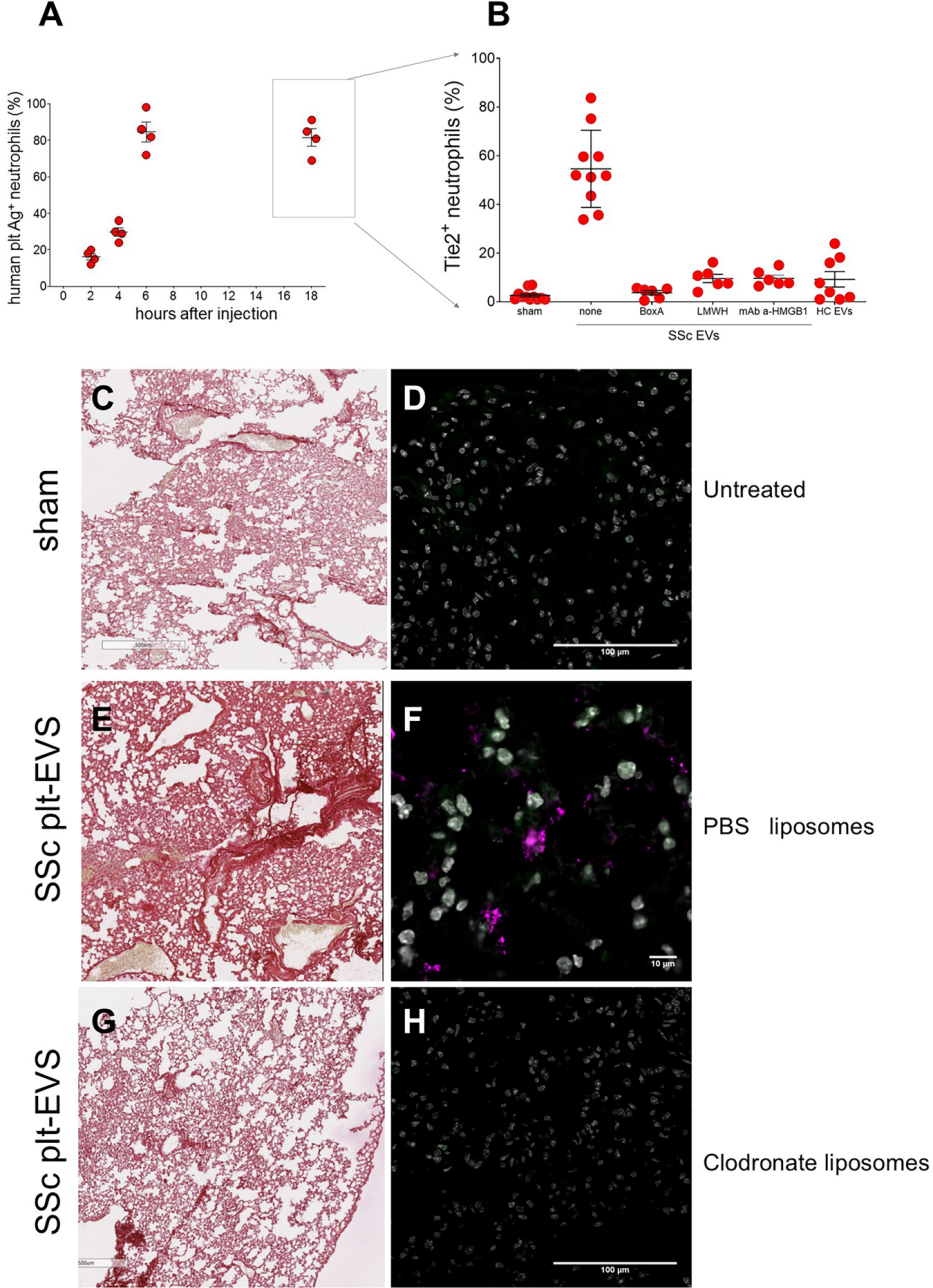
Neutrophils mediate SSc-HMGB1+ EVs-induced inflammation and fibrosis. EVs from SSc patients and controls were injected into the tail vein of NSG mice. **A** The binding of human platelet-derived EVs to neutrophils (% on y-axis) was assessed over time (hours, x-axis) post-injection. **B** Neutrophil TIE2 expression (y-axis) was measured 18 h post-injection in control (sham, *n* = 11; 6 females and 5 males) mice, in mice injected with SSc platelet-derived EVs (SSc plt-EVs, *n* = 10; 5 females and 5 males), and in mice receiving SSc plt-EVs together with HMGB1 inhibitors (Box A *n* = 6, 3 females and 3 males; LMWH *n* = 6, 3 females and 3 males; monoclonal antibody against HMGB1 *n* = 6, 3 females and 3 males). A control group of mice was injected with EVs from healthy control plasma (HC plt-EVs, *n* = 8; 4 females and 4 males) **C**–**H** Lungs were collected post-euthanasia from sham-treated control mice (**C**, **D**) and mice injected with SSc plt-EVs. Comparisons were made between mice pre-treated with saline liposomes (PBS liposomes, **E**, **F**) and those pre-treated with clodronate liposomes to inhibit neutrophils (**G**, **H**). **C**, **E**, **G** show histological analysis with Sirius Red staining. Color intensity quantified by ImageJ was 6208 in (**C**), 87270 in (**E**), and 26150 in (**G**), while **D**, **F**, **H** depict confocal microscopy findings. Neutrophils are shown in green, TIE2 in Alexa 647 (pink). DNA was counterstained with Hoechst (white).

EVs purified from the plasma of SSc patients were injected into the tail vein of immunodeficient NSG mice, which are receptive to grafting of human cells and tissues. SSc-derived, CD61^+^ plt-EVs adhered to host circulating neutrophils, with a substantial proportion (84.5 ± 5.3%) of EV-neutrophil aggregates detectable in the blood 6 h after injection (Fig. 5A). The interaction persisted over time, with most circulating neutrophils remaining associated with EVs 18 h after injection. Importantly, neutrophils interacting with SSc EVs selectively upregulated the expression of TIE2, and this event was abrogated by HMGB1 inhibitors, Box A, LMWH and monoclonal anti-HMGB1 antibodies (Fig. 5B). Adhesion to neutrophils was negligible when we injected into NSG mice EVs purified from healthy subjects, that expressed very limited amounts of HMGB1 (Fig. 3A). Furthermore, neutrophils of mice injected with EVs from healthy donors failed to upregulate TIE2 expression following the injection of EVs (Fig. 5A, B). Importantly, when experiments were performed in both male and female NSG mice, no significant sex-related differences were observed in neutrophil activation, TIE2 upregulation, or lung fibrotic remodeling induced by SSc-derived EVs (Fig. 5B). Taken together, these data indicate that EV-driven neutrophil reprogramming occurs similarly in male and female mice under the experimental conditions used. Consistently, no significant sex-related differences were observed in lung collagen deposition or neutrophil infiltration following SSc-EV injection (Supplementary Fig. 4).

After the injection of SSc-EVs, TIE2-expressing neutrophils infiltrated the lung parenchyma, resulting in alveolar obliteration and diffuse fibrotic remodeling associated with tissue injury. In contrast lung sections from the saline-treated (sham-treated) group displayed no evidence of ongoing inflammatory responses, alveolar damage, or interstitial fibrosis (Fig. 5C–F).

Selective neutrophil stunning using liposome-encapsulated clodronate, which interferes with early neutrophil migration, cytokine and chemokine production, and generation of NETs [40] prevented inflammatory changes and subsequent lung damage and remodeling in mice injected with SSc-EVs. Importantly, the lung disease elicited by SSc-EVs was unaffected in mice injected with liposomes containing saline, confirming that the effect of EVs in vivo was mediated via their action on neutrophils (Fig. 5E–H).

## Discussion

In this study, we demonstrate that neutrophils in patients with SSc exhibit distinct features of aberrant activation and autophagy, which may be integral to the disease’s progression. Notably, the neutrophil reprogramming induced by SSc-derived EVs was comparable in male and female mice, supporting the generalizability of this mechanism. Neutrophils play a role in responding to vascular injury by aiding in endothelial repair and clearing harmful agents. Under normal conditions, their activity subsides once the causative factor is resolved and the tissue has healed. However, when initial damage fails to resolve, persistent and excessive activation may occur, perpetuating vessel damage through sustained generation of inflammatory molecules and the destructive effects of NETs [17].

In SSc, a vicious cycle of inflammation, tissue injury, and NET formation may stem from the inability of blood vessels to properly repair themselves [41]. This impaired repair likely involves dysregulated angiopoietin/TIE2 receptor signaling between activated endothelial cells, perivascular cells, and potentially inflammatory leukocytes. Vasculopathy in SSc occurs independently of the extent of fibrosis and significantly contributes to morbidity [17]. Neutrophils are likely involved in the initiation and maintenance of the persistent and widespread microvascular involvement observed in SSc vasculopathy, as suggested by findings in other conditions [17, 42, 43]. However, despite mounting recent evidence involving neutrophils in SSc pathophysiology [11, 16, 44–47], there is still limited detailed data regarding their actual roles. Our findings provide new insight into this gap, demonstrating a widespread reprogramming of neutrophils in consecutive patients with SSc.

The reprogramming observed in neutrophils from SSc patients prompted us to investigate the stimuli driving this phenomenon. EVs were a promising candidate, since they could interact with neutrophils in the circulation, influencing their interaction with the microvasculature. EVs accumulate in the blood of patients with SSc [7, 13, 15, 20, 39, 48–50], possibly because of defective phagocytic clearance of P-selectin expressing substrates [14]. Most of EVs in SSc derive from platelets [13, 51] and are biologically active [7, 15, 52–55]. The connection between platelet activation, EVs accumulation, vascular injury and self-perpetuating vascular damage remains elusive.

We observe that a pivotal feature associated to the biological activity of EVs is the fact that they contain and present to the cells they interact with HMGB1, a potent mediator involved in vascular inflammation and tissue fibrosis [56, 57]. HMGB1 has been previously shown to mediate inflammatory events, fibrosis included, through its receptor RAGE in a manner which is sensitive to the environmental redox balance [58], and we hypothesized that HMGB1^+^ EVs may be responsible for neutrophil activation in SSc.

Indeed, purified HMGB1^+^ EVs induced hallmark features of neutrophil metabolic and functional reprogramming. These included granule content redistribution, autophagy, and TIE2 expression, all of which recapitulated the features observed in SSc patient neutrophils. Together, these findings indicate that activation of the HMGB1⁺ EV–neutrophil axis is sufficient to trigger neutrophil-driven lung injury in vivo. Moreover in vivo, ex vivo, and in vitro findings consistently demonstrate that SSc-derived EVs trigger NET formation—a process dependent on HMGB1–RAGE interaction—highlighting the disease specificity and mechanistic relevance of this pathway in neutrophil activation.

Recombinant HMGB1 and the RAGE agonist, glycated albumin, similarly induced neutrophil activation, suggesting that the HMGB1-RAGE axis may be a critical driver of this process. EVs purified from the plasma of SSc adhered to circulating neutrophils, with a persistent association which resulted in the selective upregulation of TIE2 on neutrophils. Blocking HMGB1 with inhibitors abrogated this response, confirming the central role of HMGB1 as a bioactive moiety presented by patients EV and responsible in driving TIE2 expression. Additionally, neutrophils from *Rage*⁻/⁻ mice, which lack the receptor for HMGB1, did not upregulate TIE2 in response to HMGB1+ EVs, whereas they remained responsive to the RAGE-independent agonist, IL-8. This further underscores the critical role of RAGE in mediating HMGB1’s effects on neutrophils.

Notably, injection of SSc-derived EVs into NSG mice led to TIE2-expressing neutrophils infiltrating the lung parenchyma, where they caused significant alveolar obliteration and fibrotic remodeling. By “stunning” neutrophils with clodronate-containing liposomes [40], we prevented these events, confirming the importance of neutrophil infiltration in mediating lung damage.

While our findings demonstrate the pathogenic contribution of HMGB1⁺ EVs in neutrophil activation and lung injury, we did not assess their impact in established chronic models of SSc. The present study was designed to define a mechanistic pathway and to demonstrate its sufficiency to induce lung pathology, rather than to model chronic disease progression. Future studies using chronic disease models will be necessary to determine whether targeting HMGB1⁺ EVs can modify disease onset, progression, or recurrence.

Overall, our findings suggest that HMGB1^+^ EVs play a central role in driving neutrophil autophagy and activation in SSc. The upregulation of TIE2 on neutrophils and the subsequent infiltration into tissues contribute to the fibrotic and vascular manifestations of the disease. This study provides new insights into the mechanisms of neutrophil activation in SSc and identifies potential therapeutic targets to mitigate neutrophil-mediated tissue damage. Further research is needed to explore the therapeutic potential of targeting HMGB1 or TIE2 pathways in SSc. Of importance a role for RAGE in SSc associated vasculopathy has already been suggested, and high levels of the soluble molecule in patients with SSc at baseline may be used to predict new onset of pulmonary arterial hypertension and to predict lower survival [59]. Understanding the precise mechanisms by which platelet-derived, HMGB1^+^ EVs interact with neutrophils and other immune and mural cells in SSc could open avenues for the development of novel targeted therapies.

## Supplementary information

Supplementary Figures

## Data Availability

All experimental data that support the findings of this study are available in open.science@unisr.it.
