# Supplementary material for: Neutrophil reprogramming underlie vasculopathy and lung disease in systemic sclerosis
Source: Cell Death Dis. 2026 Apr 4;17(1):453. doi: 10.1038/s41419-026-08690-5 (PMC13184140; doi:10.1038/s41419-026-08690-5)
Supplement: Supplementary file 1 — Supplementary Figures [file 41419_2026_8690_MOESM1_ESM.pdf]

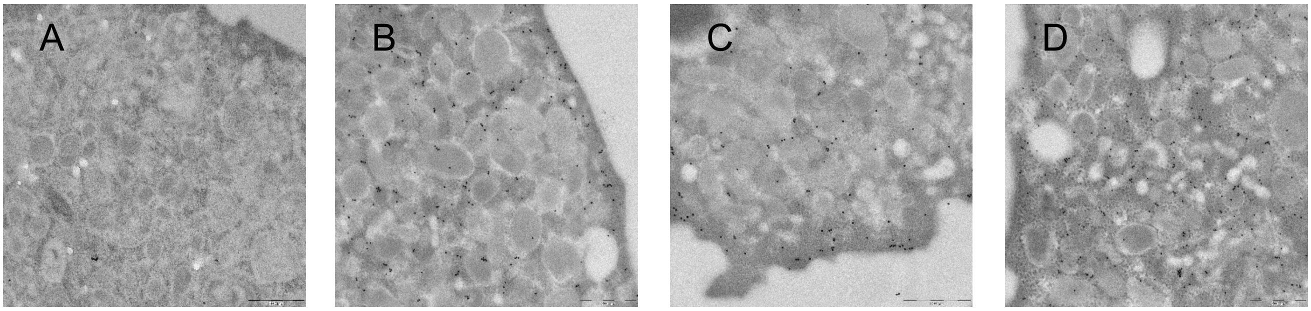

Ultrastructural localization of TIE2 on neutrophils by immunogold electron microscopy.

TIE2 expression in neutrophils was assessed by immunogold labeling and transmission electron microscopy in a representative healthy control (panel A) and in SSc patients (panels B–D). Gold particles (10 nm), indicated by arrows, mark TIE2 expression primarily along the plasma membrane and, in some cases, on intracellular vesicles. Images are representative of findings from ≥3 individuals per group. Due to the low-throughput nature of immunogold EM, no quantitative analysis was performed; for TIE2 quantification, see flow cytometry data in Figures 1 and 3.

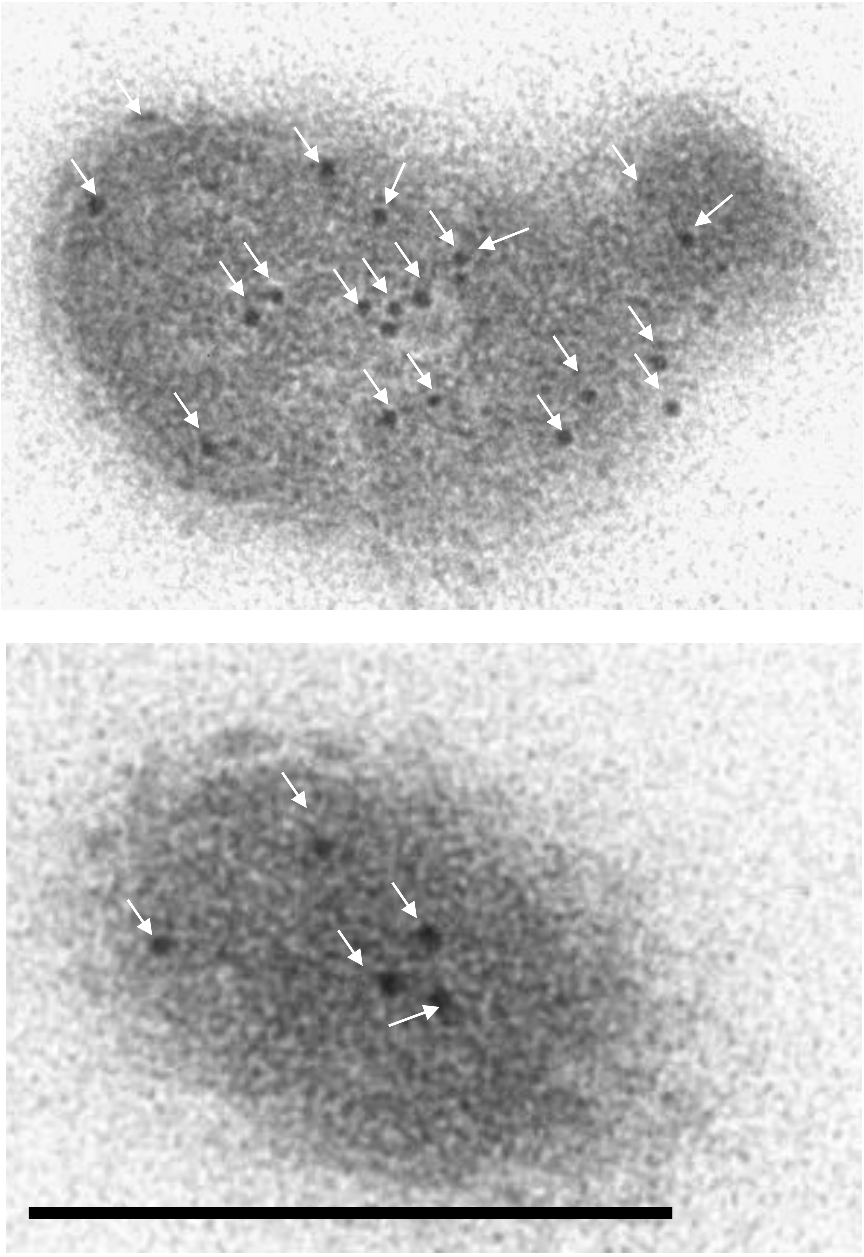

Immunogold electron microscopy of HMGB1 expression on platelet derived extracellular vesicles Gold particles (10 nm), highlighted by arrows, indicate HMGB1 localization . Images are representative of ≥3 independent experiments. Due to the qualitative nature of immunogold EM, no statistical quantification was performed.

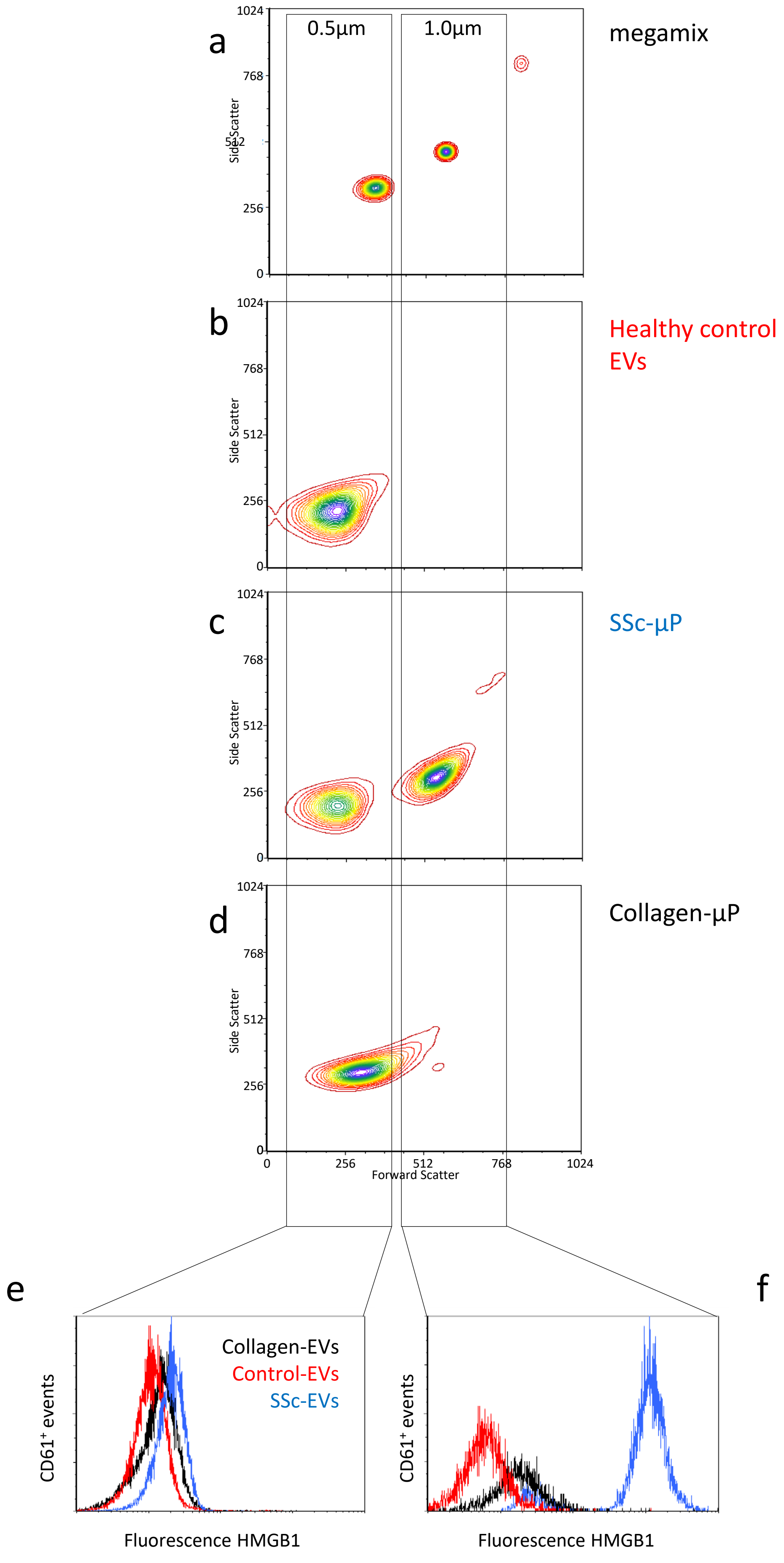

Contour plots of: (a) Megamix SSC® reference beads for size gating (0.5 and 1 µm). Following representative contour plots of platelet-derived EVs retrieved from the plasma of healthy volunteers (b, healthy control-EVs), of patients with SSc (c, SSc-EVs) and of EVs generated *in vitro* after platelet stimulation with collagen (d, collagen-EVs). Profiles in panels (e) and (f) refer to the expression of HMGB1 in the two populations of EVs identified based on their size in healthy subjects (control-EVs, red profiles), patients with SSc (SSc-µPs, light blue profiles) or generated *in vitro* after platelet stimulation with collagen (collagen-EVs, black profiles). Representative immages of EVs retrieved from different donors.

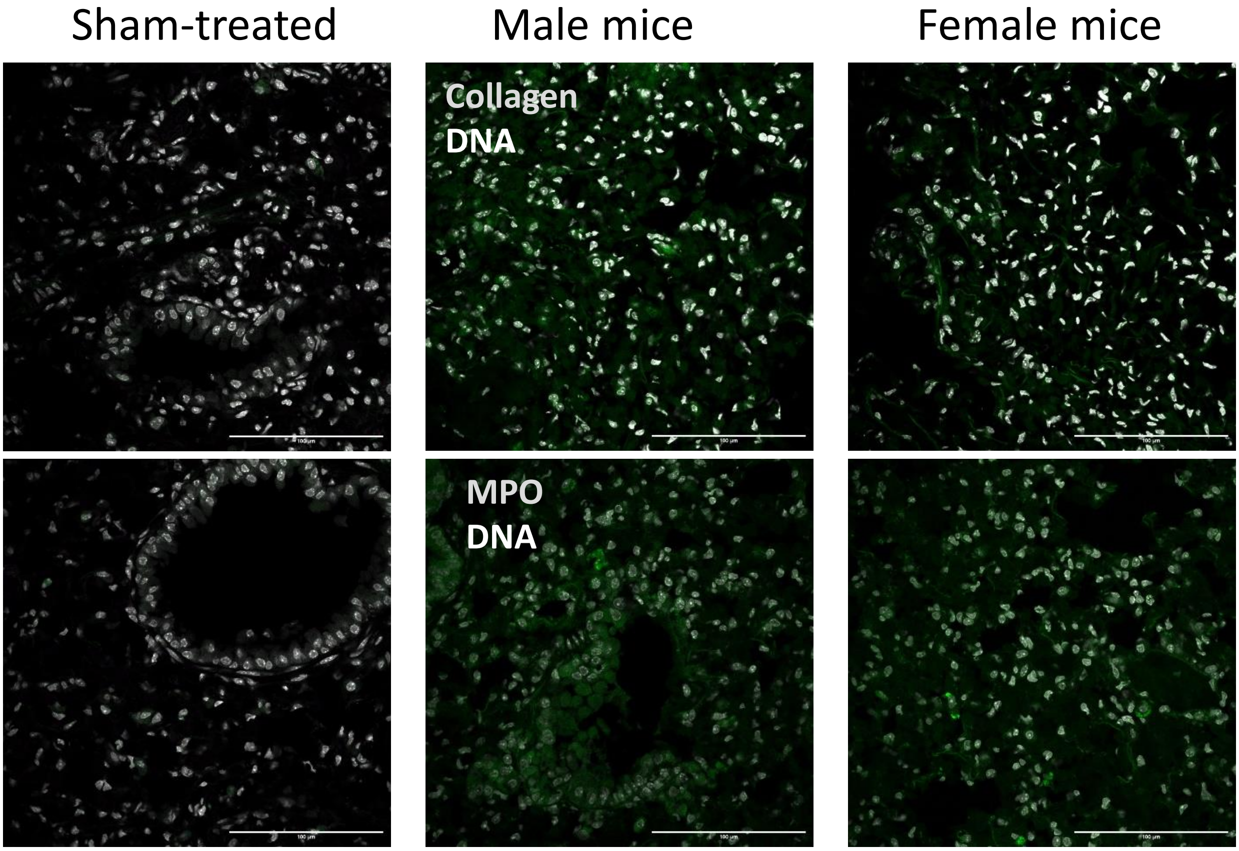

Supplementary Figure S1: Sex does not affect SSc-EV–induced neutrophil infiltration or lung fibrosis. SSc-EVs were injected into the tail vein of NSG mice, while sham-treated mice received vehicle alone. After 18 hours, lungs were collected for histological analysis. Collagen deposition was assessed using FITC-conjugated anti-collagen antibodies (green, top panels), while neutrophil infiltration was evaluated by FITC-conjugated anti-MPO staining (green, bottom panels). In all cases, DNA was counterstained with Hoechst (white). Representative images from male and female mice are shown. No significant sex-related differences were observed in collagen deposition, neutrophil infiltration, or neutrophil response to SSc-EVs.
